# A Lump in the Throat
*A Bristol University post-graduate lecture.


**Published:** 1937

**Authors:** E. Watson-Williams

**Affiliations:** Surgeon-in-charge, Ear, Nose and Throat Department, Bristol Royal Infirmary


### A LUMP IN THE THROAT.*

BY

E. Watson-Williams, M.C., Ch.M., F.R.C.S.,

*Surgeon-in-charge, Ear, Nose and Throat Department, Bristol Royal Infirmary.*

---

"A Lump in the Throat" is a complaint by no means rare. I am not now considering acute conditions, but a symptom that—though often dated from some acute illness—has usually been present and perhaps increasing in severity for some weeks or even months before advice is sought. It is definitely so described, the patient commonly indicating the cricoid region, and is frequently the only complaint. Or if accompanied by aching or other symptoms, the latter do not include hoarseness, dyspnœa, or pain on swallowing; and while there may be some difficulty in "swallowing nothing" or the saliva, there is no interference at all to the passage of food or drink—indeed, a meal is often found to relieve the symptoms.

This "lump" is important because it often causes distress out of all proportion to the seriousness of the cause. The terror of cancer is at the back of this—or even in the forefront. Cancer of the throat has a peculiar horror for most people, even those who might

* A Bristol University post-graduate lecture.

accept a diagnosis of cancer in other regions with resignation. When indirect laryngoscopy shows no obvious abnormality, and there are no suspicious neck glands: when the complaint is of "a lump" without hoarseness, or pain or difficulty in swallowing, whatever else is said the patient should always be told definitely at the first opportunity that there is nothing to suggest the presence of cancer. An astonishing number of patients show at once that this fear has been lurking in their minds, though they have given no hint of it, and are far removed from the imaginative or "neurotic."

Examination of the larynx and hypopharynx by the indirect method is obviously the first step to take. Although rare, gross organic swelling does occasionally produce this symptom. A gumma in the base of the tongue, a pharyngeal thyroid gland or an innocent neoplasm may be given as examples: malignant disease in this region almost invariably produces early pain or dysphagia, but if not it will be obvious by the time a "lump" is felt. Hoarseness is rarely absent in tuberculous laryngitis: the form which begins as œdema of the epiglottis is sometimes the cause of a feeling of a lump without other local symptoms, but it is a lesion of rather late pulmonary disease and the condition is manifest on examination of the larynx. To show how similar are the complaints of a patient with an actual swelling to the types described below I may cite those of a recent case: "For some months past I have had a lump in the throat: there is no pain, or difficulty in swallowing, but it feels all the time as if I was going to cry and didn't want to"—she had a mucous cyst of the epiglottis the size of a cherry. Such cysts, even when they are as big as this, seldom give rise to symptoms.

In the absence of an obvious tumescence we have to decide what is causing the feeling of a lump. And it is possible to recognize three fairly distinct types: although no doubt in many cases two or more conditions are concerned in producing the symptoms, and the practitioner may have to decide whether all are important, and which require treatment.

*The Lump which Moves Up and Down.*—This is especially common after an attack of influenza, with accompanying pharyngitis; but may occur independently, though usually in patients obviously in somewhat poor health. The lump is not felt during a meal, but there is often difficulty or discomfort in swallowing spittle, with a constant desire to swallow—and with each "swallow" the lump is felt to move. This symptom is nothing more than consciousness of the movement of the cricoid cartilage against the back of the pharynx during swallowing, a sensation that is normally not observed but which is obvious if anyone concentrates on the part during swallowing. Attention has been directed to the throat by the discomfort of pharyngitis, and this is continued when the hyperæsthesia would ordinarily have subsided.

*Treatment.*—In addition to reassurance, local treatment is directed to rendering the part less sensitive. An astringent lozenge may be ordered, such as Troch. Acid Tannic or Troch. Krameriæ: if at the beginning this is not well tolerated, Krameria lozenges containing Cocaine Hydrochloride gr. 1/40 may be given for a few days. A mixture containing Iron with Quinine or Nux Vomica is often helpful.

*The Lump with Aching* is most frequently encountered in women. At first it is noticed only when the patient is tired, later it is constant. There is no difficulty in swallowing: or at most the patient may find difficulty in *starting* swallowing, especially with dry food. For the throat is often "dry." Complaint may be made of "sore" throat, but on closer inquiry it is found that aching is meant, and usually the sternomastoid muscles are indicated. On examination the mucous membrane of the posterior wall of the pharynx is conspicuously pale and thin, so that the vessels in the submucosa are prominent: the mucous membrane of the nose shows the same changes, and the inferior turbinals are small, permitting the posterior wall of the naso-pharynx to be seen through the nostrils. The condition is associated with defective thyroid activity, and other evidence of this, including goitre, may be found. *Treatment* must be directed to the general condition: as a rule only a small dose of thyroid substance is required, but it may have to be continued over a long period. In the early stages local treatment also may be needed. A sedative lozenge which stimulates the flow of saliva is particularly useful, such as that composed of Chlorate of Potash and Borax, with one part per thousand of Cocaine Hydrochloride: this small addition appears to have a definite effect and does not bring the lozenge within the scope of the D.D.A.

In connection with this group I may mention the dysphagia-anæmia-stomatitis syndrome: not that the subjects of this complain of a "lump" in the throat, but because they do sometimes, before there is any real dysphagia, complain of discomfort or a sense of tightness there; and the syndrome may be incomplete, lacking the stomatitis or any obvious anæmia. The latter is a secondary anæmia with deficiency in hæmoglobin, and a colour index below unity. The stomatitis affects the pharynx, the inner surface of the lips, the angles of the mouth, where deep cracks may occur, and the tongue, which loses its papillæ. Massive doses of iron are necessary.

*The Emotional Lump* is traditionally associated with sorrow or pity, rather than anger or fear, and is due to an actual spasm of the inferior constrictor of the pharynx. The classical "globus hystericus" is "a ball which comes up in the throat and chokes me," and there is usually accompanying inability to swallow: it is transitory and recurrent, and occurring in a patient of obvious emotional instability is not likely to present difficulty in diagnosis. It is otherwise, however, when, at the other end of the scale, we are told of a lump which is constantly present in a well-nourished, hard-working, apparently placid and intelligent middle-aged male, with sufficient insight to have made sure that there is no obstruction to breathing, speaking or swallowing: who may, it is true, fear cancer, but is certain that the lump was there before any such idea entered his head. As the condition progresses there may be aching pain, usually rather lower down behind the upper part of the sternum; and the sense of constriction in the throat may become sufficiently intense to lead to retching, though without nausea. There may occur, also, a clicking sound in the throat, audible to others, either with each expiration, or every few seconds, especially when the patient lies in bed, or holds the head in a particular position.

In many patients (possibly in all) the origin is in emotional strain, especially sorrow or pity, including self-pity: and the sufferers are the apparently unemotional, even stoical, individuals who so far from desiring sympathy, seek above all to stifle their troubles and present an unruffled face to the world. What makes matters more difficult is that these troubles are genuine: one might instance the case of a man who had served his firm faithfully and successfully for thirty years, and just when he might have expected promotion was discarded owing to an amalgamation; another who had been "let down" in an important matter by someone whom he had every right to trust; and perhaps most common of all, those who have suffered the loss of dear friends or relatives. An officer who had served unscathed for four years in the forward area in France in the Great War confided that ever since then he had been unable to read a pathetic passage in a book or to witness one on the stage without tears coming to his eyes and a *lump in the throat* (which he later recognized was just the same as the "lump" that brought him for advice), though he tried always at first to overcome and later to conceal what he regarded as an unmanly and degrading weakness. Sexual difficulties have not come within my cognizance: perhaps I am a poor psychologist.

*Treatment* of this condition demands, first, a complete examination, including laryngoscopy, or even œsophagoscopy under general anæsthesia if necessary; and every additional method of investigation, such as

X-rays, pathological tests, etc., that appears at all likely to help: it is essential to convince the patient that there is no local disease, and the detection of the cause of emotional strain, or even the explanation that this is the origin of the trouble, is a great help. Local treatment is useless, and worse. In a severe case the best remedy is a mixture containing a small dose of opium: I do not believe these patients are likely to develop a "habit," provided the mixture is used for two or three weeks only. Bromides are too depressing for use alone, but a small dose at night may be valuable: one or two "Sedobrol" tablets in a cup of hot water are excellent. If the spasm is severe the patient may carry tablets of atropine, gr. 1/50, to place under the tongue when a bad attack threatens; he is, of course, warned not to take more than one in twenty-four hours. But the key-note in this, as in other "lumps in the throat," is to convince the patient that the sensation is merely an exaggeration of a normal condition, and that there is no serious organic disease present.

### Summary.

The symptom discussed is a "lump" in the throat, without hoarseness, or pain or difficulty in swallowing food; there may be aching, or difficulty in swallowing saliva. Fear of cancer is common, and the patient should always be reassured about this as early as possible. Rarely there is an actual swelling visible by laryngoscopy, and producing exactly the same sensations. The usual finding is one or more of:—

1. Pharyngeal hyperæsthesia, with or after pharyngitis. *Treatment*, astringents.

2. Aching, dry throat in hypothyroidism. *Treatment*, thyroid gland, local sedatives.

3. Emotional spasm of constrictor (especially in the stoical). *Treatment*, explanation of causation, and, if necessary, opium and bromides.

